# The need for an empirical research program regarding human–AI relational norms

**DOI:** 10.1007/s43681-024-00631-2

**Published:** 2025-01-09

**Authors:** Madeline G. Reinecke, Andreas Kappes, Sebastian Porsdam Mann, Julian Savulescu, Brian D. Earp

**Affiliations:** 1 University of Oxford, Oxford, UK; 2 City, University of London, London, UK; 3 University of Copenhagen, København, Denmark; 4 National University of Singapore, Singapore, Singapore

**Keywords:** Human–AI interaction, Relationships, Norms, Moral psychology

## Abstract

As artificial intelligence (AI) systems begin to take on social roles traditionally filled by humans, it will be crucial to understand how this affects people’s cooperative expectations. In the case of human–human dyads, different relationships are governed by different norms: For example, how two strangers—versus two friends or colleagues—should interact when faced with a similar coordination problem often differs. How will the rise of ‘social’ artificial intelligence (and ultimately, superintelligent AI) complicate people’s expectations about the cooperative norms that should govern different types of relationships, whether human–human or human–AI? Do people expect AI to adhere to the same cooperative dynamics as humans when in a given social role? Conversely, will they begin to expect humans in certain types of relationships to act more like AI? Here, we consider how people’s cooperative expectations may pull apart between human–human and human–AI relationships, detailing an empirical proposal for mapping these distinctions across relationship types. We see the data resulting from our proposal as relevant for understanding people’s relationship–specific cooperative expectations in an age of social AI, which may also forecast potential resistance towards AI systems occupying certain social roles. Finally, these data can form the basis for ethical evaluations: What relationship–specific cooperative norms we should adopt for human–AI interactions, or reinforce through responsible AI design, depends partly on empirical facts about what norms people find intuitive for such interactions (along with the costs and benefits of maintaining these). Toward the end of the paper, we discuss how these relational norms may change over time and consider the implications of this for the proposed research program.

Social relationships of different types face distinctive coordination problems. What a well–functioning (i.e., cooperative) relationship looks like depends on the nature of the relationship: teacher–student, boss–employee, colleague, friend, romantic partner, and so on. Some of these relationships are normatively expected to be hierarchical; others are more egalitarian. Some are characteristically close and caring, whereas others are more transactional [6, 11, 33]. Some are seen as appropriate candidates for sexual interaction; in others, such behavior is forbidden. However, now that artificial intelligence (AI) is beginning to assume some of these socio–relational roles, or close analogs of them, the extent to which we will, or should, hold similar expectations in human–AI relationships is unclear.

Our theoretical assumptions are as follows. For each relationship type, as picked out by lay language categories, there exists a set of socially prescribed cooperative functions—including hierarchy, care, transaction, and mating—that relationships of that type within a given society are normatively expected to serve, or not to serve, either characteristically or under certain conditions [19], drawing on [9, 11, 32, 45, 49]. “Cooperative functions” refer to mutually beneficial, socially endorsed solutions to recurring coordination problems of social life [9, 14], and “relational norms” refer to the socially prescribed set of cooperative functions for each relationship type in a given culture. This includes the strength of people’s normative expectations regarding whether a given cooperative function (e.g., hierarchy, care) should or should not be served by relationships of a given type (e.g., friends, neighbors; [19, 21]).

Relational norms likely developed to support effective cooperation in human–human relationships across different socio–cultural environments [14]. With the rise of artificial intelligence, however, socio–relational roles traditionally filled exclusively by humans are increasingly being filled by AI. We already see AI–powered chatbots beginning to serve as assistants [15, 24], coaches/advisors [44], coworkers/teammates [43], friends/companions [36], managers [40], and even “girlfriends” [17], among other social roles. As AI–powered systems become ever more capable of sophisticated social interaction, it is crucial to consider how existing human–human relational norms may be implicated across the human–AI and human–human social landscape.

In this paper, we outline a research program designed to empirically investigate how human–human relational norms may or may not transfer to human–AI interactions. This proposed research program is part of a broader ongoing investigation into the theoretical and ethical implications of human–AI relational norms.^1^ The extent to which humans apply human–human relational norms to interactions with AI systems has several important implications for the ethical and effective use and design of such systems, as well as for their regulation. For example, both ethical guidelines^2^ and regulatory acts and directives^3^ rely heavily on principles—such as transparency, safety, and privacy—to analyze the obligations of AI developers and deployers. However, the implications of these principles (e.g., what privacy demands, or even what counts as privacy) cannot be analyzed in the abstract, because they will differ across types of relationships. Just as ‘respect for privacy’ means something different in close friendships or family relationships than it does in formal employment relationships, so, too, might it mean different things for an AI romantic ‘partner,’ AI therapist, or AI work assistant. Current efforts to establish and implement regulatory and ethical frameworks for the use and development of AI are not yet sensitive to systematic variations in cooperative expectations across different socio–relational roles.

Moreover, understanding how relational norms transfer to AI could have profound implications for AI design across various domains. There are entire categories of AI systems—for example, in medical and educational AI—that rely on assumptions about appropriate interactions between AI systems and humans (e.g., students, patients, and healthcare professionals), which should be informed by a better understanding of human–AI relational norms. Such an understanding will also be relevant for successfully navigating the integration of AI systems in workplaces. For example, if studies show that people tend to overshare personal information with AI systems they perceive as friends (versus colleagues, say), organizations wanting to deploy ‘friendly’ AI chatbot assistants might need to implement stricter data protection measures or include specially tailored user education programs to avoid such perceptions and tendencies. More generally, the ability of AI systems to interact with humans in a safe, intuitive, and effective manner will depend, to a significant extent, on the ability of these systems to follow appropriate relational norms. Here, we propose that a first step toward determining which norms *are* in fact appropriate (i.e., ethically justified and pragmatically workable) for human–AI interactions across various social roles is to *empirically assess* the existing norms as judged by human users.^4^

Taking this into account, we proceed as follows: We begin by introducing the human–human relational norms model as developed by [21]. Next, we outline several theoretical reasons why we expect human–AI relational norms will likely differ, in some areas, from human–human relational norms. We also speculate about human–AI relational norms in cases of superintelligent AI—hypothetical future AI systems that can outperform humans across a variety of tasks. Drawing on this background, we propose an empirical research program to investigate human–AI relational norms. Finally, we describe necessary future steps and discuss potential implications and limitations of this research program.

## Relational norms in human–human relationships

Successful cooperation in some human relationships, such as those between parents and children, close friends, or long-term romantic partners, is typically characterized by a strong, secure attachment style and mutual adherence to the logic of care. The purpose of the *care* function is “securing basic welfare needs through non-contingent provision—or acceptance—of help or support” [21], see also [9, 11]. Successful cooperation in other relationships (e.g., between strangers) does not require strict adherence to a care-based norm, but instead may be characterized by tit-for-tat reciprocity, fulfilling an exchange-based or *transaction* function [19, 21, 45]. Additionally, some relationships function best when there is asymmetric authority between cooperative partners (*hierarchy* function), whereas others distribute power uniformly. Finally, some relationships are seen as eligible to serve a *mating* function, aimed at sexual fulfillment and/or the recruitment and maintenance of an intimate partner [9, 21].

These different functions combine in different ways, and with different strengths, in each type of relationship as picked out by lay language categories in a given society (e.g., teacher–student, boss–employee, teammate–teammate, friend–friend). For example, in [19], a representative sample of US Americans judged that a cooperative relationship between siblings should be characterized by strong norms of care, weak norms of transaction and hierarchy, and strongly negative norms for mating. This creates a distinctive, four–dimensional relational norm profile that distinguishes the sibling relationship from, say, the boss–employee relationship (i.e., weaker expectations of care with stronger expectations of transaction and hierarchy), the romantic partner relationship (i.e., stronger expectations of care and mating), or indeed any other. In turn, these relational norm profiles enabled precise out–of–sample prediction of moral judgments concerning actions, within the context of each relationship, that fulfilled or frustrated the relevant norms. Refusing to follow reasonable orders, for example, was seen as worse in relationships with stronger hierarchy norms (e.g., boss–employee), whereas failing to meet someone’s emotional needs was seen as worse in relationships with stronger care norms (e.g, friend–friend). Finally, the relational norms model was better at predicting such moral judgments than other common ways of characterizing relationships in the literature, such as by genetic relatedness, social closeness, or interdependence [19, 21].

## Relational norms in human–AI relationships

Human–human relational norms may not straightforwardly map onto human relationships with AI for several reasons. There are strong reasons to assume—as we do here—that, at least for the foreseeable future, AI systems are not and will not become sentient [10]. Consequently, we assume that AI systems lack several properties of great moral significance, such as interests, desires, and moral status. This assumption has several important implications for how cooperative functions might apply in human–AI relationships.

Consider the cooperative function of care. In humans, each individual in a care–based relationship is normatively expected to meet the other’s needs (i.e., significant welfare interests) to the best of their respective abilities (for qualifications, see [11, 45]). But in most current theories, AI–powered systems do not have welfare interests or needs. How will this affect human expectations about appropriate behavior with an AI friend or companion? Or consider the transaction function. This involves keeping track of benefits given or received to ensure fair and proportional treatment [11, 45]. But in what sense can an AI system truly be benefited—and how does the presence of a third party, the developer or deployer of the system, affect the sense of transaction? Similar concerns arise for mating and hierarchy. One possibility is that humans will intuitively be inclined to treat an AI system in a way that is consistent with the social role it has been programmed to fulfill: for example, by responding compassionately to an AI companion’s *simulation* of having a need, or by following an “order” from an AI supervisor. But there may also be some hesitation or confusion. For example, recent empirical evidence suggests that people may act as though AI systems have desires (e.g., for money) while explicitly endorsing the opposite [42].

This raises the question: Might humans apply (or misapply) relational norms that govern human–human relationships to analogous human–AI relationships? We consider this question, alongside an empirical proposal, in the remainder of the paper.

### A caveat about types of AIs

When discussing human–AI relational norms, it is crucial to distinguish between different types and capabilities of AI systems. Our research program focuses primarily on AI systems capable of simulating or performing roles traditionally performed only by humans. This excludes many current AI applications, such as music recommendation algorithms which, while technically AI, do not engage in complex social interactions that would invoke relational norms (however, see [12], for discussion).

Currently, the most relevant AI systems for our purposes are large language model (LLM)-based chatbots, such as OpenAI’s ChatGPT, Anthropic’s Claude, and Alphabet’s Gemini series. While these systems perform at remarkably high levels across a variety of domains, they are still associated with a number of limitations [3, 8, 48, 52]. However, given the rapid development of AI, any research program exploring human–AI interactions must be forward–looking, accounting for systems which are generally comparable to human performance across most tasks (“artificial general intelligence”) and, perhaps more speculatively, systems that consistently and significantly *exceed* human performance (“superintelligence”).

Superintelligent AI has been defined as machines “that surpass all the intellectual activities of any man, however clever” [28, p. 33]. This dovetails with other definitions of superintelligence, such as having the ability to achieve complex goals in complex environments [27, 41]. Though this threshold extends beyond the capacities of current AI systems, it is likely that such capacities will eventually emerge [46]. One possibility is that human–superintelligent AI relationships will mirror human relationships across key features. For example, one feature of superintelligent AI—in contrast to “narrow AI”—is its autonomy, such that it will act as an autonomous agent, engage in unsupervised learning, and set its own goals [23]. Whether people’s expectations regarding autonomous humans will transfer towards autonomous AI remains unknown.

There is also the possibility that people will *not* transfer human–human relational norms towards AI of various types. When thinking ahead about the prospect of superintelligence, there are common concerns about AI replacing—or even exterminating—humans [7, 26]. In light of potential value misalignment between human and AI interests, people may take a cautious perspective towards cultivating relationships with increasingly more powerful AI systems (perhaps especially for hierarchical roles with the AI in a dominant, rather than subordinate, position). But beyond people’s potential fear of AI [18], there may be other factors affecting their application of relational norms towards AI systems. For instance, people might differ in how they represent the capacities of AI at various stages of development (e.g., concerning whether it has humanlike interests). If someone considers an AI to be a close friend with needs and subjective experiences, then they may apply care–based norms differently than someone who denies that AI has these capacities. In the next section, we detail an empirical proposal for identifying how human–AI relational norms might differ from human–human norms in specific relational contexts.

## Empirical proposal

Whether, or to what extent, people apply human–human relational norms towards human–AI relationships is first and foremost an empirical question. Once we have a grasp of how people *do* apply these norms, we will then be in a position to undertake an ethical analysis of whether—based on candidate normative frameworks—they *should* apply them in the way(s) that they do. There might also then be implications for how AI systems designed to fill certain socio–relational roles should be designed: for example, to potentially counteract some of the more problematic ways in which humans (mis)apply human–human relational norms to human–AI relationships of different types. A better understanding of human–AI relational norms will also bear on the wider regulation of AI systems.

With this in mind, we propose a research program that builds on existing methods (see [19, 21]) to shed light on how people represent and respond to human–AI relationships. In particular, we call for adapting these methods to study “relational norms” within the context of human–AI interaction, focusing on a subset of relationship types that potentially apply to AI systems, whether now or in the future. In the original work, researchers examined moral judgments across a wide range of human–human relationship types, including long–term romantic partners, parents and children, siblings, strangers, close friends, boss-employee, acquaintances, extended family members, work colleague/classmates, roommates/housemates, teacher–student, political party members, friends–with–benefits, doctor–patient, teammates, and neighbors.

Clearly, however, not all of these relationship types have plausible analogs in human–AI relationships, even if the AI is superintelligent. For example, it is difficult to imagine an AI system serving in the role of a parent, sibling, or extended family member (at least for the foreseeable future). Therefore, we propose that researchers focus on a subset of relationship types that are most relevant to human–AI interaction, based on the functions and roles that AI systems are most likely to serve in the coming years. These might include relationships like work colleagues, supervisor–assistant, teacher–student, ‘romantic’ companion (e.g., Replika), or non-romantic friends.

### Probing human–AI relational norms

For studies in this area, we propose that researchers first provide participants with clear definitions and examples of the theorized cooperative functions (i.e., care, transaction, hierarchy, mating), ensuring that all participants apply the same concepts in their evaluations (see [20]). In the core block of a study, participants might rate the extent to which they endorse behavior in a given relationship (consisting of either two humans, or one human and an AI) conforming to a specific cooperative function (e.g., care). For example, in the case of a teacher–student relationship, researchers might present contrasting cases: e.g., a human teacher and a human student, or an AI teacher and a human student. By asking questions such as, “How important is it for a [human / AI] teacher to offer support and encouragement to a human student?”, researchers can begin to disentangle whether care–based relational norms differ across human and AI cases for that relationship type.

Analogous questions would be asked in relation to the other cooperative functions (e.g., questions about showing deference or authority in the case of hierarchy; behaving in a flirtatious manner in the case of mating, and so on), thereby filling out the four-dimensional relational norm “profile” for the given relationship—for both human–human and human–AI dyads—as judged by human participants. This would then be repeated for the full set of candidate relationships, with either an AI or human stipulated to occupy each respective role, and questions about behavior being asked in both directions (e.g., for the teacher–student example, this would mean examining not only how a human or AI teacher should act toward a human student, but also how a human student should act toward a human or AI teacher with respect to care, hierarchy, transaction, and mating).

If solicited from representative samples of a given population (e.g., citizens of a country, members of a cultural group or geographic region), the ratings collected by this method would represent (within a given confidence level and margin of error, depending on sample size) the explicit population-level “relational norms” for human–human and human–AI relationships for that population.^5^ (We discuss potential differences in relational norms *between* populations or cultural groups in a subsequent section.)

As a next step, researchers could then generate hypotheses about how these relational norms might shape, e.g., emotional responses, data-sharing tendencies, perceived ease of use, or moral judgments across different types of human–AI relationships. These hypotheses could then be tested out–of–sample. Using the example of moral judgments, for instance, researchers could evaluate how the measured human–AI relational norms in a given population predict moral judgments from another sample of participants (drawn from the same population) in relation to various actions described as taking place within a set of human–AI relationships. Specifically, researchers might prompt a new set of participants to evaluate a series of vignettes describing AI behaviors that either uphold or violate a given cooperative function. In the context of a human–AI “friendship” or “companionship,” for example, one norm–violating behavior might be that the AI companion downplays the human’s legitimate emotional needs: say, by teasing the human or dismissing their concerns when the human expresses sadness. Alternatively, a norm–upholding behavior might be something like the AI companion offering emotional support and encouragement during a difficult time. Both of these behaviors center on the cooperative function of “care.”

This is to say that, for each action and relationship, participants would rate the act on some kind of measure of moral judgment (e.g., moral wrongness/blameworthiness, goodness/praiseworthiness). From this, researchers could then determine whether people’s moral judgments reflect the previously established explicit population–level relational norms about how humans and AI should engage with one another within a given socio–cultural context. If people maintain differing relational norms for humans and AI (as compared to human–human relationships), this suggests that what is perceived as appropriate or inappropriate behavior in one case may not translate to the other case. This could shed light on why people react differently, for example, towards identical actions performed by human and AI agents (e.g., [5]). Researchers might also consider further measures probing people’s attitudes toward AI behavior (e.g., whether the agent acted intentionally, severity of the norm violation, and so on). Finally, the same sorts of questions could be asked about human behavior toward an AI. For example, it could turn out that participants believe humans should act in a “caring” manner toward an AI friend or companion, even if they deny that the AI has welfare interests (e.g., to avoid practicing behaviors that would be wrongful if done to humans); although, the strength of the expectation or judgments of the moral wrongness of violating the norm might be weaker than if the the target were a human rather than an AI.

We also see it as valuable to better understand why *individuals* may represent human–human and human–AI relationships differently from one another. For example, people’s attitudes regarding substratism—whether they have “prejudice against AIs based on their non-biological (i.e., silicon-based rather than carbon-based) material”—appear related to the tendency to consider AI as a moral patient [47, p. 3]. Further, people may differ in anthropomorphizing AI (e.g., ascribing sentience; [13, 29]), which may also affect how they represent human–AI relationships. Indeed, if an individual believes that an AI system has its own welfare, then this will likely affect whether they endorse care as appropriate for humans to direct towards AI. As such, these kinds of individual differences may be important to consider when evaluating the cognitive mechanisms underpinning human–human and human–AI relational norms.

The research design described above can be adapted to further address mechanism: For example, researchers might consider manipulating the way that AI systems are themselves described (e.g., in terms of current or predicted future capacities, anthropomorphic qualities). Alternatively, researchers might depart from the self–report metrics described above, instead implementing various behavioral measures to test people’s applications of relational norms to human–AI cases (e.g., gauging how people may speak differently with a human versus an AI companion). These kinds of comparisons across measures could clarify whether people’s explicit beliefs about how relational norms should govern human–AI relationships align with their implicit attitudes and actual behavior.

Taken together, we see this kind of empirical project as an initial step towards understanding (and perhaps even forecasting) the nature of human–AI relationships. We see this as essential for informing the design and governance of AI systems that are more trustworthy, ethical, and aligned with human values.

## Discussion and looking ahead

In this paper, we sketched how relational norms may differ between human–human and human–AI relationships across a range of contexts. We anticipate that some relational norms will transfer between human–human and human–AI relationships, whereas others will not. At present, we expect that people may be largely resistant to norms endorsing (costly) care–based behavior or mating–related behavior in human–AI relationships, although perhaps to different degrees depending on the relationship (e.g., stronger norms against flirtatious behavior when the AI is programmed to mimic a supervisory relationship than when it is programmed to serve as a ‘romantic’ companion). People may also resist being under the authority of an AI, complicating social roles characterized by hierarchy (e.g., between a manager and an employee). Indeed, people may even fear AI occupying these kinds of social roles [18] or reject that they should be in them altogether. We argue that understanding these nuances—by applying a relational norm framework that considers human–AI interaction in the context of specific social roles—is an important open direction for research in AI ethics and cognitive science, more broadly.

There is also a possibility that human–human relational norms will shift as social interaction with AI becomes increasingly common. For example, based on their increasing familiarity with AI systems in particular roles, will humans come to expect “one-way” relationships with other humans, where only one partner is acknowledged to have any needs? Will human–human relationships typically characterized by asymmetrical hierarchy norms, such as the teacher–student relationship, become more egalitarian? By studying human–human and human–AI relational norms (and, ideally, behavior) longitudinally, we can trace how these social landscapes may change over time.

Evaluating these norms longitudinally can also inform AI design. When people maintain similar relational expectations between human–exclusive and human–AI relationships, they may be more amenable to interacting with AI as a social partner, or regard those interactions as more authentic. We speculate that, at present, these expectations may be most similar in the case of common, relatively low–stakes AI social roles, such as AI assistants. But as AI models become more intelligent, capable, and humanlike in their presentation, people’s expectations regarding which social roles are appropriate for AI may evolve accordingly. Social roles for AI that currently seem far–fetched (or even terrifying, to some), such as AI as long–term romantic partner or employer, may become more plausible—and more prevalent—as these systems improve.

In addition, we see this research program as potentially highlighting unintended consequences or risks of applying human–like relational norms to AI systems, such as the risk of undue anthropomorphism or emotional manipulation [24]. By observing trends in relational norms over time, we may better predict how people will engage with future AI models, enabling us to anticipate and hopefully avoid certain types of risk. This would require developers to draw on these insights to inform responsible AI design.

Our research program will also have significant implications for interpreting or adapting AI regulation (in both the present and in the future). For example, the EU AI Act takes a risk–based approach to AI governance, classifying entire categories of AI systems as “high– or low–risk” by area of application. Thus, all AI systems applied for medical or educational uses are automatically considered high risk. Yet, it is not only the area of application that is relevant for determining risk—some uses of AI in medicine or education will be more risky than others. Future regulations informed by, and sensitive to, human–AI relational norms are likely to do a better job of identifying and addressing such risks.

Similarly, much soft law and scholarship in the area of AI ethics focuses on principles—such as safety, transparency, and privacy—which the design, development, and use of AI systems should respect [35, 37, 51]. However, as mentioned previously, how these abstract notions should be interpreted will depend on the socio–relational role the AI system is occupying and the way humans interact with it. Thus, a better understanding of human–AI relational norms may be one way of getting beyond abstract debates about principles and closer to addressing practical problems with direct impacts on human users of AI systems.

In addition to studying these norms longitudinally, we also recommend that this research be conducted cross–culturally. Advances in artificial intelligence may be not only transformative but radically transformative, leading “to societal change comparable to that precipitated by the agricultural or industrial revolutions” [30, p. 4]. These kinds of transformations affect the entire world, including future generations. Having a comprehensive understanding of human–AI relational norms for individuals of different backgrounds (e.g., within and outside of “WEIRD” cultures; [1, 2]) may be an essential step towards designing AI systems that operate properly within (i.e., with due sensitivity to) diverse socio–cultural contexts.

Importantly, relational norms or associated cooperative expectations may not only differ across countries/cultures but also within them (e.g., by demographic factors, such as age, previous experience with technology, previous experience with AI). These differences may have important implications for how AI systems are designed in light of relational norms. For example, AI systems designed to satisfy or adhere to the care function may have to exhibit very different types of caring behavior towards children, the elderly, and middle–aged adults.

In this paper, we have focused on how a better understanding of human–AI relational norms could be used to improve governance and use of AI systems. However, we recognize that this information, like conversational AI systems more generally, may be subject to dual–use risks [38]. Understanding how humans relate to AI systems differently based on social role could be harmfully misused. For example, AI systems involved in human–AI care–based interactions could conceivably be designed to elicit personal information from the human relational partner, which could then be misused in various ways [31]. One straightforward example would be eliciting financial information, such as passwords or credit card numbers; other examples include eliciting sensitive personal or medical information that could be used for targeted advertising, further model training, or sold to third parties [39]. Similarly, understanding how humans apply hierarchy–based norms to AI could be exploited to create AI systems that unduly influence human decision–making or behavior (e.g., in political contexts). Finally, there is also the risk that detailed knowledge of human–AI relational norms could be used to create more convincing deepfakes or AI impersonation systems. By mimicking the relational norms that humans expect in specific social contexts, malicious actors could create AI systems that are increasingly effective at deceiving humans in social engineering attacks or by spreading misinformation [25]. We see these as significant concerns, but we also note that understanding the dual–use potential of increased knowledge about human–AI relational norms is itself necessary to combat nefarious use cases.

While we are convinced that our proposed research program will provide valuable descriptive insights into human–AI relational norms, it is important to be clear about the distinction between descriptive findings and normative conclusions. Empirical data can inform, but cannot on their own determine, ethical prescriptions. The type of research we propose here offers a descriptive foundation upon which future normative work can build: We cannot make properly nuanced arguments about how things should be without first knowing how they are. AI systems have the potential to either reinforce or challenge existing norms, raising important ethical questions about which norms should be preserved or modified as human–AI interactions become more prevalent. However, again, such questions cannot be answered in the abstract. They require the type of empirical work we propose here.

Moreover, it is crucial to recognize that relational norms are not static but can change over time, often in response to evolving social values and practices. A prime example of this is the shift in the doctor–patient relationship over the past century.^6^ This relationship has moved from a paternalistic model, where doctors made decisions with little patient input, to one emphasizing patient autonomy and shared decision–making. What was once seen primarily through a lens of hierarchical “care” has incorporated stronger elements of patient self–determination. Such changes over time illustrate that while empirical data on cooperative functions in human–AI relationships are necessary, they do not themselves fully determine the extent to which these functions are normatively desirable.

To navigate the complex interplay between empirical findings and normative conclusions in this domain, we and other researchers can employ methodologies that bridge the descriptive–normative gap. Earp et al. [20] propose several strategies for leveraging empirical data in the service of normative arguments within experimental philosophical bioethics. These strategies include assigning *prima facie* normative weight to widely–shared moral judgments among a group of stakeholders, if these judgments can be shown to be robust and appropriately responsive to normative reasons; using “debunking” strategies to subtract normative weight from moral judgments that are fickle or inappropriately responsive to normatively (ir)relevant factors (e.g., framing effects, but see [16]); and assigning comparable normative weight to diverse views among different groups of stakeholders, if these views can be shown to track different, yet reasonable underlying ethical commitments (i.e., adopting a policy of pluralism). All of these could be applied to research into human–AI relational norms. Complementing this approach, Savulescu et al. [50] propose and defend the Collective Reflective Equilibrium in Practice (CREP) procedure, which integrates empirical data on stakeholder preferences and expert judgments with ethical principles to generate justified policy recommendations.

Ultimately, the empirical research program proposed here must be complemented by both “armchair” ethical theorizing in philosophy, as well as the types of integrative methodologies just mentioned. Nevertheless, we argue that it is crucial for all of these approaches to inform each other, and to be based on a rich descriptive understanding of the relational norms that people in different socio–cultural contexts do, in fact, hold—even if it is ultimately argued that some of these norms have undesirable consequences (or are intrinsically problematic) and should therefore be reformed or abandoned.

## Conclusion

In the present work, we have argued that there may be considerable value in understanding how people represent (and distinguish between) human–human and human–AI relational norms. As AI systems advance and continue to assume socio–relational roles traditionally filled by humans, it will be critical to understand how these developments may complicate people’s cooperative expectations about relationships—whether that be with other humans, or, indeed, with AI. Given the rapid pace of AI development, we think it is crucial to explore human–AI relational norms and translate these findings into contextually–sensitive AI governance and design. We expect that changes in human–AI relational norms will track with advances in AI models; we therefore urge policy–makers and scholars in this area to help facilitate and keep abreast of developments in research on, and understanding of, human–AI relational norms.

Relationship–specific cooperative norms governing human–AI interactions represent a critical and largely unexplored frontier in AI ethics, governance, and design. By examining how people apply, adapt, or resist applying human–human relational norms in human–AI contexts, we can inform more nuanced AI development, governance, use, and integration strategies. The potential implications of this work are far–reaching, from improving the design of AI systems to informing more contextually–sensitive and effective AI regulations. Moreover, by tracking how these norms evolve over time and across cultures, we can better understand, prepare for, and influence the long–term societal impacts of widespread human–AI interaction. We call upon researchers across disciplines, policymakers, and AI developers to engage with this crucial area of study. Only through collaborative, interdisciplinary efforts can we hope to navigate the complex landscape of human–AI relationships.

## Footnotes

1 A more comprehensive theoretical framework exploring the ethical and philosophical dimensions of human–AI relational norms is currently being developed by the authors and colleagues. The empirical research program outlined in this paper is designed to complement and inform this theoretical work.

2 See, e.g., UNESCO’s Recommendation on the Ethics of Artificial Intelligence [51].

3 Such as, e.g., the EU AI Act [22] or the US Executive Order on the Safe, Secure, and Trustworthy Development and Use of Artificial Intelligence [4].

4 It will also be important to study how AI systems themselves represent, adhere to, or depart from human–human relational norms when interacting with human users. This could be accomplished both by explicitly asking AI systems the same or similar questions to the ones we propose for human participants, or by asking an AI system (e.g., GPT, Claude, LLaMA) to pretend it is occupying a particular socio–relational role with respect to the human user, measuring various aspects of its behavior, and then repeating this paradigm over multiple sessions while systematically manipulating which role the system is asked to occupy or mimic.

5 It could also be interesting to infer participants’ *implicit* relational norms, for example, by assessing their reactions to various types of behavior as expressed within different relationships: After all, people may sometimes *believe* or *report* that they endorse certain norms (e.g., around gender egalitarianism) while intuitively reacting in a way that suggests they hold different norms (e.g., feeling distressed by women’s expressions of autonomy). As an aside, the examples just given point to a different type of norm (i.e., *gender norms*) that may interact with relational context (e.g., different expectations around childcare for mothers as compared to fathers). As our proposed research program develops, it will be important to “fill in” the social identities of the agents described as occupying different social roles, including their gender, racial or ethnic identity, class membership, ingroup/outgroup status, and so on, to see how these features interact with relational context to drive normative expectations for what constitutes appropriate or inappropriate behavior in a given society. This is especially relevant insofar as AI systems designed to fill certain types of social roles (e.g., assistant, carer) may be programmed to reflect prevailing—and potentially biased or unjust—norms around gender, race, etc. For a recent critical discussion of the tendency of moral psychologists to study “raceless, genderless strangers,” see Hester and Gray [34].

6 We thank an anonymous reviewer for this key point and example, which we adopt with gratitude.
